# O16 A diagnosis of BehÇet’s Disease with secondary antiphospholipid syndrome

**DOI:** 10.1093/rap/rkab067.015

**Published:** 2021-10-19

**Authors:** Alexandra Jones, Lucy Paterson-Brown, Vinay Shivamurthy

**Affiliations:** Evelina London Children's Hospital, London, United Kingdom

## Abstract

**Case report - Introduction:**

Behçet’s disease (BD) is a systemic inflammatory, variable-vessel vasculitis manifesting most commonly with recurrent oral or genital ulcers and uveitis. The heterogeneous presentation may also include skin, joint, gastrointestinal and central nervous system involvement. There is a geographical prevalence in countries along the “Old Silk Road” and a strong association with HLA-B51 antigen. Paediatric-onset BD commonly has an incomplete clinical picture thereby causing a diagnostic challenge.

We present the case of a 10-year-old boy of Lithuanian origin whose history, imaging and results led to a rapid diagnosis which is unusual in the paediatric population.

**Case report - Case description:**

The patient presented with a 1-month history of abdominal pain, distension and a cough that did not respond to oral antibiotics or inhalers from his GP. His mother recalled swollen proximal interphalangeal joints on both hands, a rash and red eyes approximately 4 days earlier which had now resolved. She also reported mouth ulcers occurring at least once a month from a young age and cold sores without any genital or anal ulcers. He also had a history of reactive arthritis at the age of 3 with bilateral knee swelling and anaemia.

He was afebrile, tachycardic with respiratory distress and an oxygen requirement to maintain saturations over 94%. Chest X-ray showed large bilateral pleural effusions and cardiomegaly. Echocardiography confirmed a large pericardial effusion with signs suggestive of tamponade physiology.

After admission to PICU he was intubated and sedated to facilitate pericardial and pleural drain insertion. Post-procedure he was haemodynamically stable.  Empirical antibiotic therapy was commenced with IV ceftriaxone.

Investigations showed a CRP 58mg/L, ESR 37mm/hr, LDH 325U/L, Haemoglobin 98g/L, platelets 223x10^9^ and a blood film showed non-specific changes. All microbiology samples were negative including T spot, mantoux and broncho-alveolar lavage. Serology included negative Anti-Nuclear Antibody, Extractable Nuclear Antigen, double stranded-DNA, and Rheumatoid Factor with normal complements, immunoglobulins and serum amyloid. HLA B51 was reported as positive. Faecal calprotectin was high at 539µg/g Antiphospholipid antibody screen was positive (anticardiolipin antibody 68U/mL, Beta2-glycoprotein 111U/mL). CT demonstrated multiple pulmonary emboli (PE), splenomegaly and moderate volume ascites. Ophthalmology assessment was unremarkable and biopsies taken during oesophago-gastro-duedenoscopy and colonoscopy were normal.

He was diagnosed with BD and secondary antiphospholipid syndrome (APS) based on a combination of clinical, radiological and blood markers. Treatment included colchicine as monotherapy, diuretics to manage effusions and dalteparin for the PEs.

**Case report - Discussion:**

The Consensus Classification of Paediatric Behçet’s Disease (2015) requires 3 out of 6 of the following features to diagnose BD; recurrent oral ulcers, genital ulcers, skin involvement, ocular inflammation, neurological signs and vascular abnormalities.  Our patient would score 3 out of 6, thereby meeting criteria for diagnosis.

The extensive vascular findings on CT are in keeping with variable vessel vasculitis. Antiphospholipid antibodies including Anticardiolipin (aCL) antibeta- 2 glycoprotein I (B2-GP1) and Lupus anticoagulant (LA) are observed in autoimmune diseases including BD, with around 2% of secondary APS cases on the Ped-APS registry being associated with BD. The frequency of vascular involvement is reported to be 5—20% in paediatric series with deep vein thrombosis (DVT) and superficial vein thrombosis (SVT) being most common. The combination of antibodies and levels are important in assessing risk of thrombosis.

There is no difference in the acute treatment of thrombosis attributable to APS as compared to other causes. Current guidelines following thrombosis with persistently positive antiphospholipid antibodies is for long-term coagulation. Children are most commonly transitioned from low molecular weight heparin to warfarin for long-term anticoagulation with a target INR of 2-3 following the first thrombotic event. Direct oral anticoagulants (DOACs) are not currently recommended pending further evidence. Recent studies include an RCT in adults with APS which was halted early due to excessive arterial thrombotic events in the rivaroxaban arm and in another study, rivaroxaban didn’t reach the non-inferiority threshold. However, recent EULAR recommendations regarding APS treatment in adults concluded that DOACs may be considered in certain settings.

Colchicine was chosen as initial therapy based on presentation with history of mucocutaneous involvement and arthritis. Despite widespread inflammation there were no ocular or neurological findings suggesting the need for anti-TNF-a as first line therapy.

**Case report - Key learning points:**

Paediatric BD is still a poorly understood condition and presents diagnostic challenges. This case highlighted the importance of a thorough history including a full systemic enquiry, without which the diagnosis would have been more difficult. Once the effusions were drained and the patient was started on colchicine, his follow up echocardiograms returned to normal and he has only reported one much smaller mouth ulcer in the last 4 months. Uveitis screening has continued without any clinical concerns. At this stage there is no clinical need to progress to second-line treatment.

The patient developed significant needle phobia during his intensive care admission, so DOAC therapy was considered for long-term anticoagulation for the APS, given the lack of need for INR monitoring. However, it was decided to continue on warfarin due to the aforementioned international paediatric guidance.

